# Vocal paresis as an early or underrecognized manifestation of Post-Polio Syndrome

**DOI:** 10.1016/j.bjorl.2026.101818

**Published:** 2026-04-22

**Authors:** Gustavo Polacow Korn, Juliana Paulino do Amaral, Monique Medeiros de Moura Barreto Alves, Rosiane Yamazaki, Karlo Nunes, Hendrick Henrique Fernandes Gramasco, Noemi Grigoletto de Biase

**Affiliations:** a Universidade Federal de São Paulo, Setor de Laringologia e Voz do Departamento de Otorrinolaringologia e Cirurgia de Cabeça e Pescoço, São Paulo, SP, Brazil; b Universidade Federal de São Paulo, Setor de Neurofisiologia Clínica do Departamento de Neurologia e Neurocirurgia, São Paulo, SP, Brazil

## Introduction

Postpoliomyelitis Syndrome (PPS) is a condition that can occur several decades after the initial poliomyelitis infection.^1^ While dysphonia or vocal fatigue are common manifestations, in some cases, it may also present with dysphagia, new-onset fatigue, vocal fold atrophy, and respiratory distress.^1^^,^^2^ Paralysis or paresis of the vocal folds in patients with a history of poliomyelitis is rare, with only a few cases described in the literature. We present a case in which the patient's vocal complaints prompted him to seek medical attention, ultimately leading to the diagnosis of Postpoliomyelitis Syndrome. A written informed consent was obtained from the patient for publication.

## Case report

A 62-year-old retired male patient (former metalworker) presented with intermittent hoarseness for 4-years, which exacerbated with the consumption of cold foods, but without associated complaints of dysphagia or dyspnea. He also reported weakness in his right lower limb and cold intolerance. His past medical history included Systemic Arterial Hypertension (SAH), Acute Myocardial Infarction (AMI) in 2023, Benign Prostatic Hyperplasia (BPH), and poliomyelitis during early childhood (approximately 1-year and 6-months of age), which resulted in motor sequelae primarily affecting the left lower limb.

**Fig. 1:**
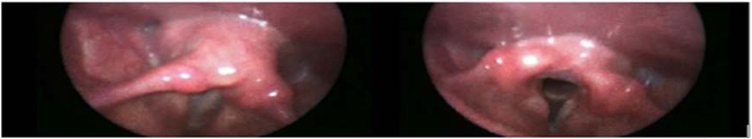
Nasofibrolaryngoscopy during alternating sniff and /i/ tasks, demonstrating decreased abduction of the left vocal fold.

**Fig. 2:**
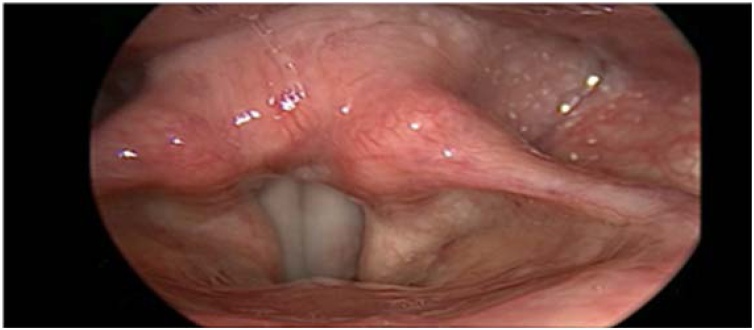
Larynx with a left paresis with the triad signals by Rapoport et al.: the interarytenoid cleft tilt toward the left side; hypercontraction of the right side; and greater ventricular show on the left side.

**Fig. 3:**
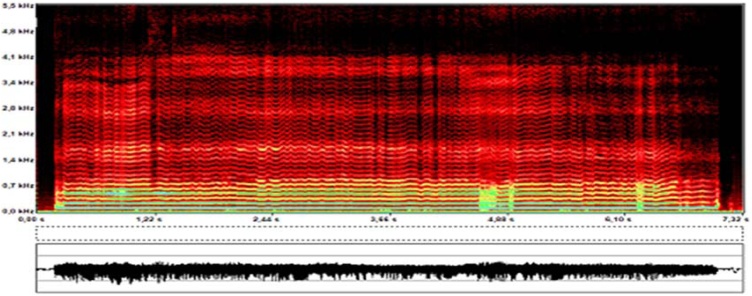
Acoustic spectrogram of the sustained vowel “é”. Note the presence of harmonics and high energy in the spectrum up to 4100 Hz, as well as the irregular morphology of the tracing.

**Fig. 4:**
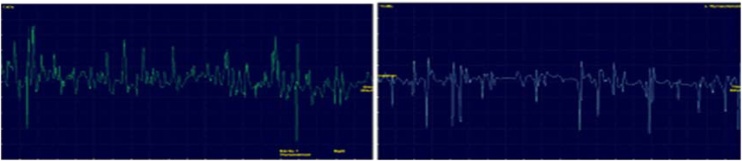
Laryngeal electromyography with electrodes in the right thyroarytenoid muscle (green trace) and left thyroarytenoid muscle (blue trace).

Laryngological evaluation revealed velopharyngeal competence but also evidence of left vocal fold paresis (Fig. 1, Fig. 2). Auditory-perceptual assessment of the voice revealed an overall grade of deviation of 2, roughness grade of 2, breathiness grade of 1, asthenia grade of 0, tension grade of 2, and instability grade of 2, according to the GRBASI scale. The CAPE-V protocol indicated an overall deviation of 70 mm, roughness of 68 mm, breathiness of 45 mm, tension of 53 mm, and instability of 50 mm. Acoustic voice spectrography of the patient revealed harmonics extending up to 4100 Hz, irregular harmonic morphology below 1000 Hz, abrupt trace interruptions due to frequency breaks, and vertical striations along the trace (Fig. 3). Imaging tests were performed to further evaluate the vagus nerve pathway, revealing no abnormalities that could account for the reported symptoms. Laryngeal electromyography revealed signs of chronic reinnervation (high-amplitude and increased duration motor unit action potentials, polyphasic, with reduced recruitment, determining a sparse interference pattern), most pronounced in the left thyroarytenoid muscle (Fig. 4). Within the clinical context and associated with similar findings in other segments (bulbar, cervical, thoracic, and lumbosacral), this finding is consistent with anterior horn involvement, characteristic of poliomyelitis sequelae. With a presumptive diagnosis of PPS, he was referred for follow-up with speech therapy and neurology. After 3-months of voice therapy, the patient demonstrated significant improvement in his vocal condition.

## Discussion

A Finnish study, which evaluated 51 patients with PPS between 2003 and 2004, reported that 29.4% of them experienced daily swallowing or voice production difficulties.^3^ According to Abaza et al., a thorough medical history and clinical suspicion of PPS should be considered in patients presenting with vocal fold paresis or paralysis.^1^

In our patient, besides hoarseness, which exacerbated with the consumption of cold foods, weakness was reported in his right lower limb and cold intolerance. These elements suggest a systemic neurological condition. Our patient exhibited laryngological findings consistent with vocal fold paresis, as described by Rapoport et al.^4^ Layngeal EMG with signs of chronic denervation may contribute to a diagnosis of a chronic neurological disease and in this case of limb involvement, a EMG evaluating other segments could contribute to finding that are characteristic of poliomyelitis sequelae.^1^ However, the absence of findings consistent with chronic denervation in laryngeal electromyography points toward an alternative diagnosis. Thus, EMG plays a crucial role in guiding clinical reasoning and establishing the correct diagnosis.

Considering PPS as a diagnostic of exclusion, imaging studies of the vagus nerve pathway are crucial for excluding other potential etiologies, including neoplastic, degenerative, and vascular diseases. A prior history of paralytic poliomyelitis with subsequent recovery, followed by the onset of new muscle-related symptoms and after exclusion of other potential etiologies, supports the diagnosis of Post-Polio Syndrome (PPS).

The patient achieved good results with speech therapy. We previously reported a case involving dysphonia and dysphagia where voice therapy proved effective.^5^ Further studies involving more cases of dysphonia related to PPS are needed. As observed in the cases reported by Abaza et al., it is possible that these patients are in the early stages of PPS, and their early identification is crucial for appropriate follow-up.^1^

## ORCID ID

Monique Medeiros de Moura Barreto Alves: 0000-0003-3435-5275

Rosiane Yamazaki: 0000-0002-7960-4143

Karlo Nunes: 0000 0001 8526 9188

## Conclusion

Vocal fold paresis may be as an early or underrecognized manifestation of Post-Polio Syndrome. A thorough medical history and a high index of suspicion for PPS should be considered when motor changes in the larynx are observed.

## Ethical considerations

An informed consent was obtained from the patient. Patient confidentiality will be maintained. No harm was done to any individual. It is an observation based on clinical practice.

## Funding

None.

## Acknowledgments

None.

## Data availability statement

The authors declare that all data are available in repository.

## Declaration of competing interest

The authors declare no conflicts of interest.
